# Fan-Shaped Complete Block on Helical Tomotherapy for Esophageal Cancer: A Phantom Study

**DOI:** 10.1155/2015/959504

**Published:** 2015-02-12

**Authors:** Chiu-Han Chang, Greta S. P. Mok, Pei-Wei Shueng, Hsin-Pei Yeh, An-Cheng Shiau, Hui-Ju Tien, Chi-Ta Lin, Tung-Hsin Wu

**Affiliations:** ^1^Department of Biomedical Imaging and Radiological Sciences, National Yang Ming University, Taipei 112, Taiwan; ^2^Division of Radiation Oncology, Department of Radiology, Far Eastern Memorial Hospital, New Taipei City 220, Taiwan; ^3^Biomedical Imaging Laboratory, Department of Electrical and Computer Engineering, Faculty of Science and Technology, University of Macau, Macau; ^4^Department of Radiation Oncology, Koo Foundation Sun Yat-Sen Cancer Center, Taipei 112, Taiwan

## Abstract

Radiation pneumonitis (RP) is a common complication for radiotherapy of esophageal cancer and is associated with the low dose irradiated lung volume. This study aims to reduce the mean lung dose (MLD) and the relative lung volume at 20 Gy (*V*_20_) and at low dose region using various designs of the fan-shaped complete block (FSCB) in helical tomotherapy. Hypothetical esophageal tumor was delineated on an anthropomorphic phantom. The FSCB was defined as the fan-shaped radiation restricted area located in both lungs. Seven treatment plans were performed with nonblock design and FSCB with different fan angles, that is, from 90° to 140°, with increment of 10°. The homogeneous index, conformation number, MLD, and the relative lung volume receiving more than 5, 10, 15, and 20 Gy (*V*_5_, *V*_10_, *V*_15_, and *V*_20_) were determined for each treatment scheme. There was a substantial reduction in the MLD, *V*_5_, *V*_10_, *V*_15_, and *V*_20_ when using different types of FSCB as compared to the nonblock design. The reduction of *V*_20_, *V*_15_, *V*_10_, and *V*_5_ was 6.3%–8.6%, 16%–23%, 42%–57%, and 42%–66% for FSCB 90°–140°, respectively. The use of FSCB in helical tomotherapy is a promising method to reduce the MLD, *V*_20_, and relative lung volume in low dose region, especially in *V*_5_ and *V*_10_ for esophageal cancer.

## 1. Introduction

Radiotherapy is an important component in multimodality treatment for patients with esophageal cancer. According to the Radiation Therapy Oncology Group (RTOG) 85-01 trial, for nonsurgical treatment, the standard therapy for patients with localized esophageal carcinoma is radiotherapy to 50 Gy plus concurrent chemotherapy for better control of local tumor and fewer distal metastases. According to the National Comprehensive Cancer Network (NCCN Ver. I.2013) guideline, factors including the area at risk for microscopic disease, setup uncertainties, and respiratory and swallow motion should be considered in the treatment planning. Thus, planning target volume (PTV) of esophageal cancer on the longitudinal direction is much longer than the gross tumor volume (GTV). However, several radiosensitive organs at risk (OARs) such as spinal cord, heart, lungs, liver, and kidneys that surrounded the PTV must be considered in the treatment planning to prevent potential complications. Sample complications include radiation pneumonitis (RP) and fibrosis in the lungs, pericardial effusion and myocardial ischemia in the heart, and myelitis in the spinal cord. Among them, RP is one of the most dose-limiting toxicities and acute complication for thoracic radiotherapy of esophageal cancer [1–5].

Asakura et al. [6] found all lung dose volume histogram (DVH) parameters significantly associated with grade 2 or higher RP and they determined the optimal threshold of relative lung volume (*V*_5_–*V*_50_) to predict symptomatic RP. Another study suggested that lung volume receiving low dose related more to the incidence of RP as compared to the lung volume receiving high dose [7]. Wang et al. [2] ensured that the incidence of postoperative pulmonary complications reduced when an adequate volume of lung was spared of radiation.

Helical tomotherapy (HT) (Tomotherapy Inc., Madison, WI) is a modality for delivering intensity-modulated radiation therapy (IMRT) treatments using a rotating linear accelerator mounted on a continuously moving slip ring gantry in synchrony with the couch motion. Image-guided pretreatment alignment is feasible through mega-voltage computed tomography (CT) scan to verify the tumor position. Compared to traditional IMRT or 3-dimensional conformal radiotherapy (3DCRT), a higher degree of dose conformity and homogeneity in the target can be achieved for HT due to larger number of degrees of freedom in beam arrangement during gantry rotations to fit the lesion slice by slice, while sparing the adjacent OARs at the same time [8–13]. Chandra et al. [13] suggested that when more beams at different directions crossfire at the tumor, the volume of normal tissue with low-dose exposure may be increased and the lateral beams that irradiated a larger lung volume should be avoided. However, standard HT using 51 gantry angles through 360° in esophageal cancer led to extensive low-dose distribution in both lungs. Although directional and complete blocks of radiation beams are feasible in HT, currently there are no clear guidelines and systematic evaluations in reduction of the lung volume with low-dose exposure for treating esophageal cancer with HT.

In this study, we proposed to reduce the lung volume with low-dose exposure in HT for esophageal cancer by “blocking” the radiation beams, that is, closing the MLC, at certain angles especially in the lateral direction to spare the exposure of OARs. The directional block was defined as avoiding only incoming radiation beams while the complete block was defined as restricting the radiation beams to enter and exit certain volume [3, 14]. The degree of blocking was further quantified by the fan-shaped complete block (FSCB) to indicate the fan angles of beam blocking.

## 2. Material and Methods

An anthropomorphic body phantom (ATOM 701; CIRS, Norfolk, VA) was scanned under a CT simulator (Discovery, GE, USA) in this study. The CT slice thickness was 2.5 mm for the entire thorax. We delineated the normal OARs and a hypothetical esophageal tumor, that is, the GTV, located at the middle esophagus of the thorax with size of 102.4 cm^3^ and longitudinal length of 12.5 cm on every CT slice using the Pinnacle treatment planning system (Version 7.6; Philips Medical Systems North America, Andover, MA, USA). The clinical target volume (CTV) was defined as the GTV extended 0.5 cm radially and 4 cm superiorly and inferiorly to cover region with subclinical disease. The PTV was defined as the CTV plus 0.8 cm margin in 3 dimensions for daily setup uncertainties and internal movement, including respiration and swallowing motion. The size of PTV is 497.73 cm^3^ with longitudinal length of 22.1 cm from apex of lung to the top of liver. Other outlined structures included spinal cord, heart, right lung, left lung, and the virtual block.

**Figure 1:**
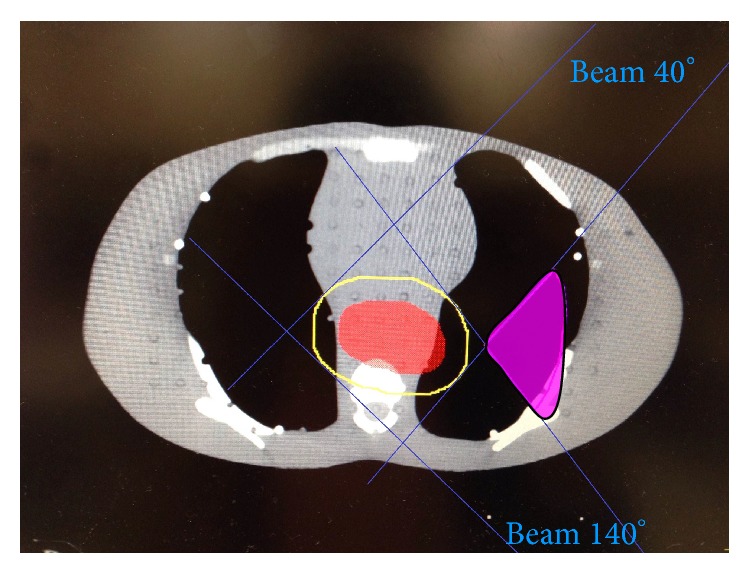
The fan-shaped block of FSCB 100° (pink area) was defined by the intersection of beam angle of 40° and 140°. The PTV was shown in red and the blue line defined the block auxiliary structure which was sufficient to cover 1.5 cm expanded from the PTV (yellow circle).

We designed 6 virtual FSCBs to form a fan-shaped block of radiation in both lungs with fan angles from 90° to 140°, each with increment of 10°. In order to avoid insufficient dose coverage at the peripheral PTV after using the FSCBs, the block auxiliary structure was sufficient to cover region expanding 1.5 cm from the PTV margin in 3 dimensions (Figure 1). For our standard phantom, we determined the 2-beam angles to achieve the fan-shaped block by the following equation for the left lung:

(1) $$\begin{array}{c} \text{Beam}\;\;\text{angle}\;\;\#1=\frac{\left(180-\text{fan}\;\;\text{angle}\right)}{2},\\ \text{Beam}\;\;\text{angle}\;\;\#2=180-\frac{\left(180-\text{fan}\;\;\text{angle}\right)}{2}. \end{array}$$

Thus, Figure 1 showed that the FSCB 100° was defined as the intersection of the beam with beam angle of 40° and 140° in the left lung. Similarly, the FSCB 100° in right lung was delineated with the beam angles of 320° and 220°.

After contouring all structures, the CT images were transferred to the HT planning system (Version 3.2.2.35 Tomotherapy Inc., Madison, WI). A prescription dose of 50.4 Gy in 28 fractions was defined for the 95% PTV. The parameters for treatment planning were field width of 2.5 cm, pitch of 0.287 [15, 16], and modulation factor of 3.0 with normal resolution mode. During the dose optimization process the dose constraints were adjusted to obtain adequate and homogeneous target volume coverage while minimizing dose in heart, spinal cord, and both lungs. The dose constraints of normal OARs were based on the RTOG 1010 and the related studies [2, 4–7] and they are summarized as follows: the maximum dose was <45 Gy for the spinal cord; the mean dose of heart was <34 Gy and *V*_40_ of the heart was <50%; the mean lung dose (MLD) must be <20 Gy; and percent volume of the organ receiving more than a threshold dose, that is, 20, 15, 10, and 5 Gy (*V*_20_, *V*_15_, *V*_10_, and *V*_5_) for lungs, was expected to be <20%, 30%, 50%, and 55%, respectively.

This study evaluated various FSCBs with different fan angles and nonblock design and investigated the correlation between the reduction of low-dose lung volume and different beam angles of FSCB. For PTV, the homogeneous index (HI) [17, 18] was defined as the ratio of minimum dose received in 5% and 95% of the PTV:

(2) $$\begin{array}{c} \text{HI}=\frac{D_{5}}{D_{95}}. \end{array}$$

A HI value closer to 1 indicates better dose homogeneity. The conformation number (CN) proposed by Riet et al. [19, 20] takes irradiation of both target volume and healthy tissues into account:

(3) $$\begin{array}{c} \text{CN}=\frac{\text{T}{\text{V}}_{\text{RI}}}{\text{TV}}\times \frac{\text{T}{\text{V}}_{\text{RI}}}{V_{\text{RI}}}, \end{array}$$

where TV_RI_ = target volume covered by the reference isodose, TV = target volume, and *V*_RI_ = volume with the reference isodose. A CN value closer to 1 indicates good target conformity and coverage.

Several dosimetric parameters including MLD, *V*_20_, *V*_15_, *V*_10_, *V*_5_ for lungs and HI and CN were evaluated for different fan angles of FSCB.

## 3. Result

**Figure 2:**
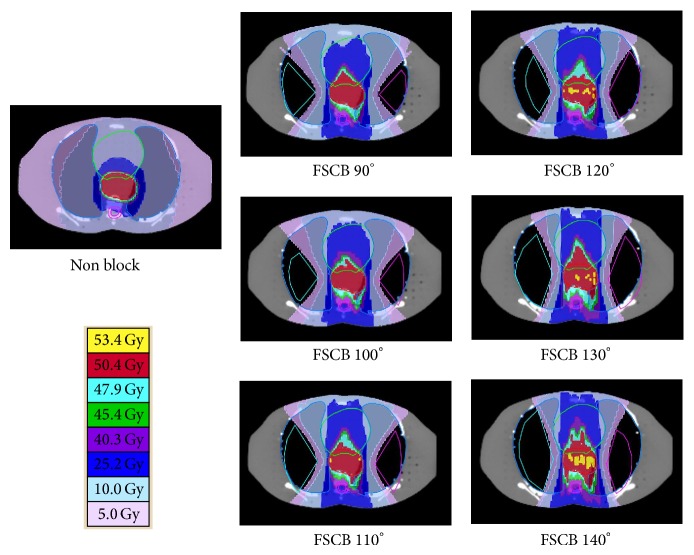
The dose distribution for nonblock and FSCB 90° to 140° designs.

The dose distribution for the seven treatment plans is shown in Figure 2. For the nonblock treatment planning, low-dose region with 5 Gy and 10 Gy spread among both lungs. For FSCB with larger fan angles, more lung volume could be protected. However, the prescribed dose would be extended to the anterior and posterior regions of the PTV. Also, the conformity and homogeneity of PTV would be degraded and the dose on spinal cord and heart was higher as compared to the nonblock design.

**Figure 3:**
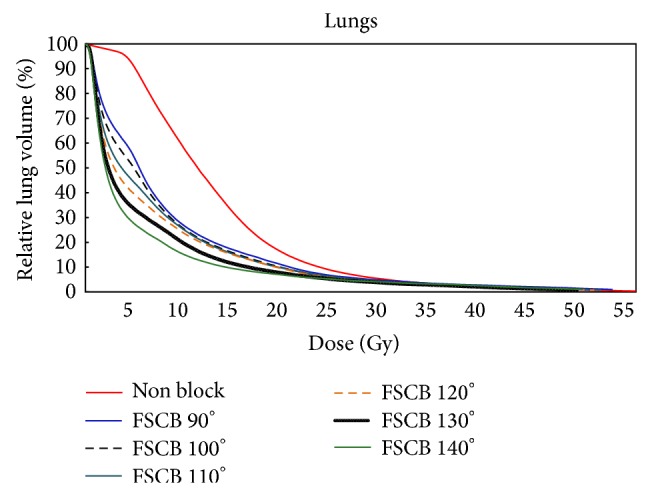
Cumulative DVHs of lung comparing nonblock and FSCB with various fan angles.

Different quantitative dose indices for the OARs and PTV were shown in Table 1. In the nonblock design, the CN and the HI were ~1.0, indicating homogenous dose distribution and idea conformation within the target volume. However, its *V*_10_ and *V*_5_ were >75%, indicating most of lung volume received low-dose exposure. The MLD and *V*_20_ from different FSCB designs were less than half of dose compared to the nonblock design. The *V*_5_, *V*_10_, and *V*_15_ for the lungs were also substantially lower for the FSCB designs (Figure 3), with simultaneous increase of the maximum dose of spinal cord, mean dose, and *V*_40_ of the heart. Their HI and CN for the PTV degraded correspondingly.

## 4. Discussion

Previous studies showed that HT provides dosimetric merits in target coverage and homogeneity, as well as better dose distribution and sharp dose gradient in reducing complication probability in OARs [10]. Our study showed that the HI and CN were good; OARs such as spinal cord and heart received relatively low dose in the nonblock treatment planning, probably due to unconstrained irradiation beam angle in rotational dose delivery. With the use of FSCB, the irradiation beam angle of HT would be restricted to protect the fan-shaped area in the lungs. Hence, when the fan angle of the FSCB is larger, a relatively larger lung volume will be protected. The reduction of lung volume irradiated in low-dose region, MLD and *V*_20_ from FSCB can potentially lead to reduced incident of RP as studies have found that the volume of lung spared from doses of 5 Gy (VS5) was the major factor associated with pulmonary complications [2]. Also, RP has been shown to be associated with *V*_20_ > 20% [21] and MLD > 13.6 Gy [22]. Thus, it is important to reduce the MLD, *V*_20_ and low-dose volume in both lungs for rotational beam delivery.

However, the maximum dose of spinal cord, the mean dose, and *V*_40_ of heart increased and the CN and HI became worse with larger fan angle of the FSCB. That was probably due to the fact that the main direction for beam delivery is either anterior-posterior (AP) or posterior-anterior (PA) position. The spinal cord is a critical organ and its maximum dose was set to 45 Gy in the treatment planning. The heart received more radiation dose in FSCB designs, that is, 9–12 Gy and 7–19% increase in mean dose and *V*_40_, respectively. However, these doses were still lower than the conventional AP:PA fields followed by off-cord oblique field technique in esophageal treatment planning.

This phantom study showed a quantitative dosimetric comparison between traditional nonblock and FSBC designs for HT. However, the optimal radiation treatment plan for esophageal cancer is actually patient-specific as it is highly dependent on the actual tumor volume. Smaller fan angle will be used for a larger tumor and less lung volume can be protected. For example, only smaller fan angles of FSCB, that is, 90° and 100°, are recommended for elliptical PTV which includes gross lymph node and elective nodal regions to assure sufficient peripheral coverage of PTV. However, it will lead to more lung dose as compared to larger fan angles. For PTV only includes gross tumor volume, larger fan angles of 110°–140° are suggested. When the PTV is not located on the body midline, asymmetric fan angle of FSCB in right and left lung should be considered. For example, when the PTV covers the gastroesophageal junction which is near the left lung, the fan angle of FSCB in the left lung would be smaller than that in the right lung. We can also adjust the location of beam angles #1 and #2; that is, beam angle #1 in left lung can be placed at the posterior lung to protect more left lung volume, if we prefer the same fan angle for both lungs. The size of the tumor volume also influences the FSCB design. Otherwise, the location of the virtual fan shape structure can be adjusted to protect more lung volume. The shape of the chest also affects the total lung volume and the fan angle of FSCB, with larger fan angle (120°–140°) for thick chest and smaller fan angle (90°–110°) for thin chest. In clinical practice, all aforementioned factors should be considered and proper fan angle can be decided for optimal tradeoffs among protected lung volume, irradiated dose to OARs and PTV dose conformity and homogeneity. With the use of FSCB, the treatment time will increases accordingly due to prescription dose deliver in restricted beam angle, that is, 14–25.6 mins for FSBC 90°–140°, while it is 8 mins for nonblock design.

## 5. Conclusion

This phantom study showed that HT with FSCB can reduce MLD, *V*_20_, and low-dose lung volume, especially in *V*_5_ and *V*_10_ for esophageal tumor. And the maximum dose of spinal cord and mean dose of heart were increased because of the incident beams from anterior and posterior direction of the helical rotation. For the FSCB plans, CN and HI in PTV are slightly worse and treatment time is longer than nonblock design plan. The evaluated FSCB fan angles of 90°–140° can cover most clinical cases and the dose reduction increased for larger fan angle. Since MLD, *V*_20_, and low-dose lung volume are reduced with FSCB designed in HT and also highly associated with RP, FSCB designed in HT is a promising protective method for reducing RP.

## Figures and Tables

**Table 1:** Quantitative dose indices for lungs, spinal cord, heart, and tumor for treatment plan of nonblock and various FSCB designs.

	Nonblock	FSCB 90°	FSCB 100°	FSCB 110°	FSCB 120°	FSCB 130°	FSCB 140°
Lungs							
MLD (Gy)	14.53 ± 0.94	9.38 ± 0.26	9.06 ± 0.11	8.73 ± 0.09	8.12 ± 0.04	7.33 ± 0.32	7.05 ± 0.04
*V*_20_ (%)	18.04 ± 0.91	11.76 ± 1.46	11.42 ± 0.47	10.70 ± 0.99	10.57 ± 0.80	10.28 ± 1.02	9.38 ± 0.13
*V*_15_ (%)	36.84 ± 0.22	21.11 ± 2.03	20.10 ± 0.77	18.85 ± 0.30	17.27 ± 0.37	14.49 ± 1.86	14.13 ± 0.27
*V*_10_ (%)	77.19 ± 4.72	34.34 ± 1.03	32.24 ± 0.16	30.31 ± 3.71	28.14 ± 1.48	21.10 ± 2.40	20.68 ± 0.26
*V*_5_ (%)	97.03 ± 0.12	54.07 ± 0.63	52.41 ± 0.80	46.74 ± 1.35	41.50 ± 0.68	33.41 ± 1.32	30.79 ± 0.19

Spinal cord							
maximum dose (Gy)	33.57 ± 0.57	41.64 ± 0.79	41.87 ± 0.64	42.86 ± 0.45	43.41 ± 0.15	44.02 ± 0.14	44.45 ± 0.10
Heart							
mean dose (Gy)	20.93 ± 0.50	29.15 ± 0.83	29.27 ± 0.36	31.03 ± 0.41	31.50 ± 1.11	32.11 ± 0.94	32.41 ± 0.37
*V*_40_ (%)	6.72 ± 0.03	13.97 ± 1.22	15.07 ± 0.94	17.60 ± 0.44	19.02 ± 3.77	23.26 ± 1.59	25.78 ± 0.50

PTV							
CN	0.91 ± 0.00	0.82 ± 0.02	0.82 ± 0.01	0.82 ± 0.00	0.77 ± 0.02	0.75 ± 0.03	0.71 ± 0.01
HI	1.02 ± 0.00	1.04 ± 0.00	1.04 ± 0.00	1.06 ± 0.00	1.07 ± 0.00	1.07 ± 0.01	1.07 ± 0.00

## Abbreviations

GTV: Gross tumor volume
CTV: Clinical target volume
PTV: Planning target volume
OAR: Organ at risk
RP: Radiation pneumonitis
FSCB: Fan-shaped complete block
DVH: Dose volume histogram
MLD: Mean lung dose
HI: Homogeneous index
CN: Conformation number
HT: Helical tomotherapy
IMRT: Intensity-modulated radiation therapy
NCCN: National Comprehensive Cancer Network
RTOG: Radiation Therapy Oncology Group
MLC: Multileaf collimator.
